# A review of recruitment and retention strategies in U.S. local health departments: insights and practical solutions

**DOI:** 10.3389/fpubh.2025.1516027

**Published:** 2025-07-17

**Authors:** Olivia Houck, Skky Martin, Harshada Karnik, Jonathon P. Leider, Gina Massuda Barnett, LaMar Hasbrouck

**Affiliations:** ^1^Center for Public Health Systems, Division of Health Policy and Management, School of Public Health, University of Minnesota, Minneapolis, MN, United States; ^2^Cook County Department of Public Health, Forest Park, IL, United States

**Keywords:** public health workforce, staffing, recruitment, retention, local public health department

## Abstract

**Introduction:**

Staffing shortages in US local health departments (LHDs) have been well documented. While the increasing number of public health graduates offers an abundant talent pool, LHDs are facing increasing competition from other employers.

**Methods:**

We conducted a comprehensive review to identify factors impeding recruitment and retention at LHDs and strategies that could be used to address them.

**Results:**

Our findings highlight various barriers and opportunities. The main barriers were non-competitive salaries, perceived lack of employee autonomy, cumbersome HR protocols, and an environment that is not satisfying to a diverse workforce. Strategies to enhance recruitment include marketing the rewarding aspects of public health employment, establishing partnerships with academic institutions, and developing internship programs. Strategies to improve retention include improving the organizational work environment, supporting professional growth, mentoring programs, and succession planning.

**Discussion:**

Our study highlights the staffing barriers facing LHDs and offers practical solutions they can implement to support successful recruitment and retention. More work is needed to identify specific ways to improve workplace culture, quantify the disparity between pay offered by health departments and their competitors, and better understand barriers and opportunities related to supporting a diverse workforce.

## 1 Introduction

US local health departments (LHDs) play a critical role in meeting evolving community needs and promoting healthy populations. The US public health workforce has historically been understaffed, even prior to the COVID-19 pandemic. Between the 2008 Great Recession and 2019, the governmental public health workforce decreased by nearly 16%, losing over 38,000 full-time equivalents (FTE). To adequately provide population health services nationwide, an estimated 80,000 additional FTE—an 80% increase—are needed (1). Although there were temporary staffing increases to address the COVID-19 pandemic, many LHD across the country reported high attrition rates due to retirements, burnout, and fatigue (2, 3). Budget cuts can also result in loss of staff, with the downstream effect of reducing services provided and clients served (4). The public health workforce declined despite a surge in the number of public health degree graduates as nearly 20,000 undergraduate public health degrees were conferred in 2020 (5) and 19,000 graduate degrees in 2016 (6). This underscores the prospective public health workforce supply of public health degree graduates that public health departments can tap into to address the imminent gaps in the workforce. It is important to understand the barriers to this workforce supply and how to effectively drive graduates into public health employment (7).

There have been numerous reviews on the state of the public health workforce conducted in the last two decades (8–10). Beck and Boulton reviewed 126 articles related to the size and composition of the public health workforce, workforce effectiveness and health impact, public health workforce demands, and public health workforce policy. Hillard and Boulton reviewed literature that focused on four public health workforce areas: (1) diversity; (2) recruitment, retention, separation, and retirement; (3) education, training, and credentialing; and (4) pay, promotion, performance, and job satisfaction. Looking specifically at their findings on recruitment and retention, Hillard and Boulton found that studies mentioned strategies such as career development, flexible work schedules including telecommuting, succession planning, scholarships, loan repayment and/or forgiveness, reduced tuition, monetary incentives and bonuses, and mentoring programs. In this study, we extend existing literature that focuses on the public health workforce shortage by reviewing public health workforce literature that solely identifies recruitment and retention barriers faced by LHDs. Based on the review, we provide strategies that LHDs can implement to strengthen their workforce.

## 2 Materials and methods

We conducted a literature review in October 2022 to identify peer-reviewed literature on LHD recruitment and retention. The purpose of the review was to survey the recent literature to provide insight into the recruitment and retention barriers that LHDs are facing and to provide strategies to strengthen the public health workforce. We adapted the Preferred Reporting Items for Systematic reviews and Meta-Analyses (PRISMA) approach (11)—a common set of guidelines for systematic reviews—for developing our protocols and conducting our searches. Our approach diverged from the PRISMA approach in two ways. First, we did not assess for heterogeneity, or potential bias because our goal was to present existing recruitment and retention programs and strategies that health departments can adapt to their own agencies as they see fit. While we did not use formal tools to assess study quality, this was a consideration during the screening process. Upon review, no articles stood out for exclusion due to poor quality. Second, we did not register our study in Prospero as required by the PRISMA protocol.

### 2.1 Search strategy

The search strategy aimed to obtain original peer-reviewed literature pertaining to LHD recruitment and retention. All articles were obtained using “Publish or Perish 7” software which records and conducts replicable queries across 3 different databases: PubMed, Google Scholar, and Web of Science. Publish or Perish is an online application developed to retrieve and analyze academic citations using a variety of data sources to obtain the raw citations, analyze these, and present a range of citation metrics (12). The files of citation metrics that Publish or Perish generates for each search include basic information about the article as well as number of citations and h-index. These files helped facilitate the compilation and review of our search results. We executed multiple searches for articles published between January 2002 and October 2022 with search terms focused on four concepts: career status (early, mid, and senior), public health workforce (i.e., public health agency, public health department, public health program, public health institution, and public health students), recruitment (i.e., public health recruitment, public health hiring, public health staffing, pathway building opportunities, and public health pipeline), and retention (i.e., public health retention, development, and workforce environment). A full table of search terms can be found in Appendix A.

### 2.2 Study selection

The following inclusion criteria were determined *a priori*: (1) peer-reviewed article, (2) implemented in the United States, (3) related to the local public health workforce (i.e., public health departments), and (4) published in English between January 2002 and September 2022. Studies related to state and federal public health workforce were to be included only if they were relevant to local public health. Duplicate articles were removed in the data collection Excel spreadsheet by one author (OH). Study selection followed a two-phase screening process with one reviewer (OH). First, titles and abstracts were screened to assess if articles were relevant based on inclusion criteria. Then, the articles’ full text were reviewed. The main reason for exclusion during screenings was because they were not focused on public health departments. We read each of the final articles in full and recorded a description and a summary of findings for each. Additionally, during full-text screen, four additional articles were obtained from peer-reviewed articles found in our search. These additional articles were added as supplementary sources in the final set of articles. Once this process was completed, we sorted the articles into overarching themes using an inductive approach.

### 2.3 Data extraction

Publish or Perish citation outputs contain metadata fields like author and year. Additional fields were based on an *a priori* data extraction list created by authors SM, HK, and OH. This list included relevance for LHDs (best practices to create a sustainable workforce pathway), article objective, and main findings. Based on data extraction, OH conducted a thematic analysis using an inductive approach to identify themes within the citations. Results were managed and analyzed using Microsoft Excel in a data collection spreadsheet.

## 3 Results

### 3.1 Selection results

**Figure 1:**
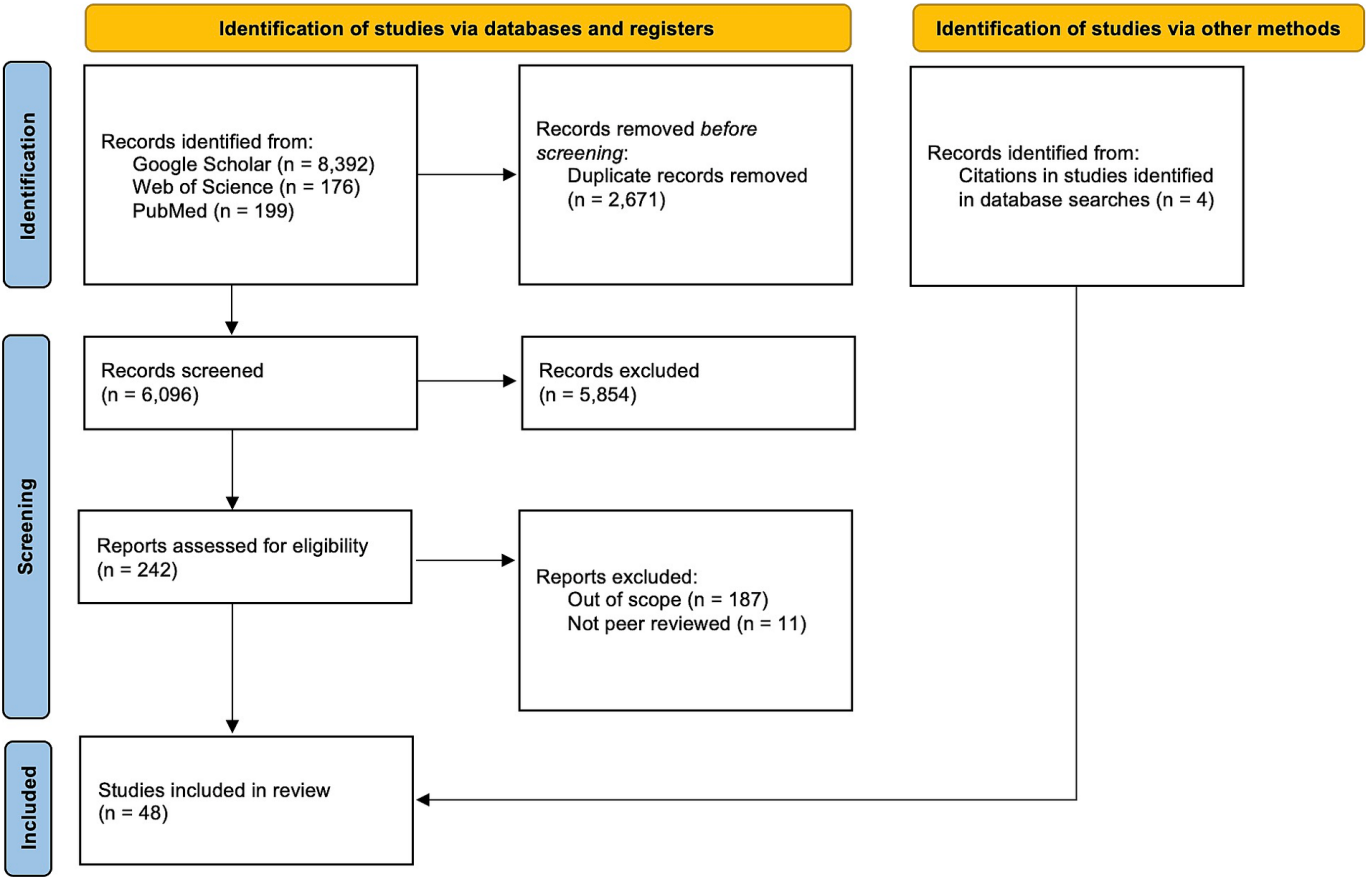
The search process.

The parallel search strategy using multiple different Publish or Perish searches of Google Scholar, PubMed, and Web of Science resulted in 8,392 articles, 199 articles, and 176 articles, respectively (Figure 1). In total, our searches yielded a total of 8,767 articles that included both peer-reviewed and gray literature. 2,671 duplicate articles were removed in Excel. 6,069 titles and abstracts were screened first to assess if articles were relevant based on inclusion criteria. 242 articles moved on to full-text review, where a further 198 were excluded. The main reasons for exclusion during screenings were because citations were not peer-reviewed and/or they were not focused on public health departments. The remaining citations (*n* = 44), along with the additional articles obtained from citations (*n* = 4) constituted our final set for full review (*n* = 48).

**Figure 2:**
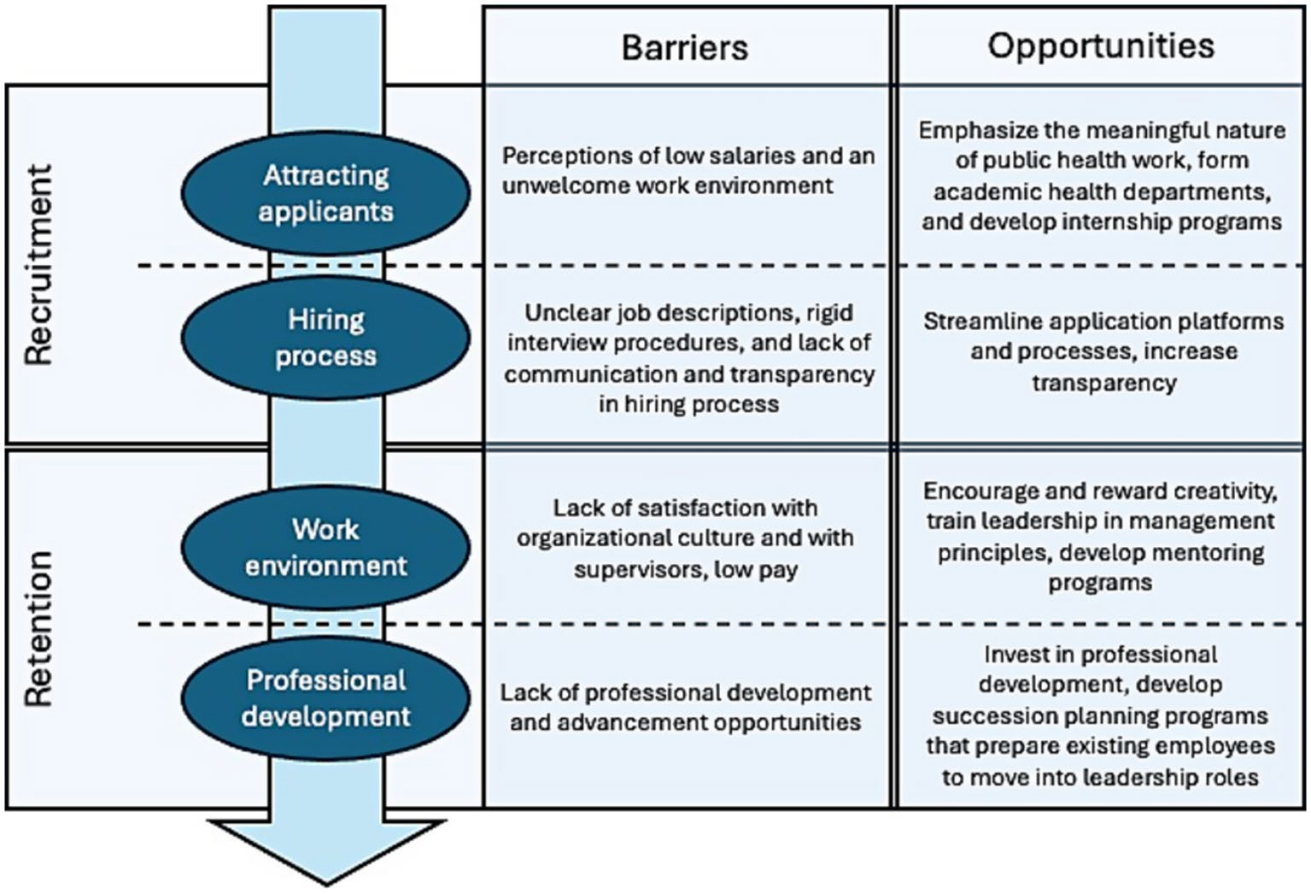
Recruitment and retention barriers and opportunities.

We observed two themes related to recruitment: attracting applicants and the application/hiring process. We also observed two themes for retention: organizational work environment and professional development. In the following sections we present barriers and opportunities for these themes. The body of articles we reviewed often included the same types of barriers. However, they varied in content on the ways to address these barriers. Thus, opportunities presented here may be more extensive than the barriers. Figure 2 provides a visual of how the results are presented.

### 3.2 Recruitment

#### 3.2.1 Attraction—getting people interested

##### 3.2.1.1 Barriers

Recruitment begins with attracting potential applicants to employment. We observed two main barriers that public health departments experienced in terms of recruitment: perceptions of an unsatisfying work environment and low salaries. Public health employment is perceived as an unsatisfying environment for diverse employees while being most friendly toward white employees (13).

Salaries that are not competitive with other employment sectors was cited as a potential reason why public health students and new graduates did not find governmental public health employment to be appealing (14, 15). One small pilot survey (*n* = 83) found that 17.8% of recent public health graduates from six schools of public health across the United States cited pay as a major deterrent to seeking employment in governmental public health (15). A qualitative study that engaged public health students and recent graduates in focus groups identified fair pay as a key component of fulfilling and meaningful work (14). Additionally, an assessment of public health job postings before and after the onset of the COVID-19 pandemic found that competition from the insurance sector has increased (16).

##### 3.2.1.2 Opportunities

Actively creating a “welcoming and satisfying” work environment for employees of all racial and ethnic groups can help foster a more diverse workforce (13). Black public health students also reported important barriers to public health employment: family financial situations creating difficulty completing degrees and accessing internships; lack of personal and family experience navigating the higher education system, and racism and stereotyping (17). Focus groups with Black public health students and graduates suggests early exposure to public health as a field for Black students to increase awareness of the field and its potential as a career path. Additionally, emphasizing how public health can help communities by addressing social determinants of health can bolster a positive impression of public health (17).

No specific opportunities were offered in the literature specifically related to improving salaries. However, there are other ways to work to offset this barrier. While LHD’s often cannot offer salaries as high as their competitors, LHDs have the unique benefit of being viewed as employers who do meaningful work (14, 15). The service-oriented mission of LHDs is a very salient factor relative to private-sector employers. In one survey, 90% of federal, state, and local public health workers reported feeling that their work was important (18). Leaning into this mission and emphasizing it in marketing efforts to potential employees can help LHDs stand out. Job-related factors like job security, competitive salaries, and benefits are also attractive to potential employees (14, 15, 18–20). Loan forgiveness also offers an excellent incentive to attract suitable applicants (14), particularly since a majority of public health graduates have $55,000 or more in debt (21).

In addition to positive marketing to attract public health talent, LHDs can also partner with academic institutions–either formally or informally–to become an Academic Health Department (AHD) (22, 23). AHDs create an exchange of skills and talent between health departments and the academic institution that benefits both parties. This can create pathways to recruit public health students to LHDs and offer opportunities for continuing education for health department staff (23). AHDs also create an environment in which LHDs can collaborate with academic institutions on public health curricula, development of certificate programs, and building pathways to public health degrees (23, 24). Health departments should also actively market toward potential applicants from disciplines other than public health, as non-public health degree holders do commonly work in health departments (25).

Student internship experiences are often a requirement in public health degree programs and thus are a common component of the public health workforce pathway. Internships can benefit the LHD by attracting future employees either directly or by supporting word-of-mouth referrals (22, 26–29). One internship program aimed specifically to undergraduate students from groups underrepresented in public health found that the program was successful in increasing their interest in pursuing public health employment (30, 31). Interns add to the workforce capacity of LHDs by contributing tasks related to data collection and analysis; program planning and evaluation; and development of reports, manuscripts, posters, and presentations (22, 26, 32). Internships also provide an opportunity for the professional development of preceptors by allowing them to exercise mentorship and management skills (26).

There are many potential benefits to providing compensation as part of internships. Compensation would help increase the pool of quality applicants (28), reduce inequity in opportunity for participation (17, 28), and make the internship more competitive with other opportunities (33). Health departments can address this by seeking potential funding sources for internship stipends. There are also existing internship programs that place interns with host sites and cover the cost of intern pay (34). Virtual internships can offer the additional benefit of internship flexibility (35).

#### 3.2.2 Application and hiring process—getting people in the door

##### 3.2.2.1 Barrier

Going beyond attracting applicants, the hiring process itself poses more barriers for those who do decide to apply. Unclear job descriptions, rigid interview procedures, and a lack of transparency around work locations, hiring processes, and timelines contribute to recruitment barriers (14, 15, 36). Potential employees cite the lack of communication that they often experience with LHD’s as a deterrent for applying for LHD positions (15). These barriers create frustration for both applicants and hiring managers, and can deter applicants from continuing with the process (14, 15, 36).

##### 3.2.2.2 Opportunities

Streamlining application platforms where possible and increasing transparency around the roles and benefits can improve the application process for potential employees (14). Characteristics of more efficient application platforms include being easily searchable, and require “minimal work on the part of applicants.” For example, using an uploaded resume to populate application fields rather than requiring applications to duplicate effort by uploading a resume and still manually filling out all application fields. Additionally, these positions should be posted to popular job sites, particularly those that allow applicants to apply directly through the platform. To make the application process more transparent, job descriptions and qualifications should be easy to understand, pay rates should be clearly posted, and a contact person should be provided (14). Additionally, to widen the applicant pool, LHDs should consider experience level should when determining minimum qualifications for a role, as practical experience can make up for less formal education (37).

### 3.3 Retention

#### 3.3.1 Organizational work environment—getting people to stay

##### 3.3.1.1 Barriers

Once new employees have been hired, a new set of barriers emerges related to getting them to stay at their specific LHD or within the field of governmental public health in general. These barriers center around the organizational work environment and offering satisfactory pay. Several parts of the organizational work environment are cited as reasons why current public health employees may consider leaving their jobs. Dynamics between supervisors and their staff is an important component of organizational culture. Satisfaction with supervisors and organizational support had a positive effect on employees no longer intending to leave their organizations (38). Such satisfaction included creativity being rewarded, being treated respectfully, feeling like the agency valued their professional development, and effective communication within the agency (38).

Unsatisfactory pay, in addition to being a barrier in attracting applicants, is also a barrier in retaining existing employees. Current public health employees who are satisfied with their pay are more likely to be satisfied with their job and less likely to consider leaving their current position (18, 19, 39–42). Among younger staff in particular, pay has been reported as the top reason for considering leaving their job (19, 20).

##### 3.3.1.2 Opportunities

There are several ways that LHDs can improve the organizational work environment to improve employee retention. This can include encouraging and rewarding creativity, implementing effective workplace communication, treating employees with respect, being supportive of employees, and fostering workplace flexibility (38, 43, 44). Engaging staff in accreditation activities is associated with an increased perception of leadership support (45). However, while public health managers may be highly trained in their areas of expertise, they are often not formally trained in the development of employees and organizations. Supporting this training for leadership can empower them to improve the work environment (19).

Providing mentoring opportunities can act as a workplace benefit to improve employees’ workplace satisfaction. LHDs nationwide have experimented with a variety of mentoring methods including one-to-one, peer mentoring, mentoring circles, speed mentoring, mentoring partnerships, online mentoring, and apprenticeship mentoring (46–48). Notably, mentorship holds exceptional value for employees from underrepresented or minoritized groups (49). To support equity in mentorship programs, LHDs have been encouraged to ensure that mentorship programs are accessible to all employees, minority employees are encouraged to participate in these programs, and that the responsibility of equitable participation rests on senior management (49). This consideration is particularly important if programs allow managers to select mentees through informal channels.

Although existing employees commonly report being unsatisfied with their pay, studies on both state and local public health employees found that they also report being happy with the benefits and job stability that comes with public health employment (20, 39). Continuing and even augmenting benefits where possible could improve both recruitment and retention efforts.

#### 3.3.2 Professional development—investing in the existing workforce

##### 3.3.2.1 Barriers

A lack of professional development and advancement opportunities within the agency are a major factor for current public health employees. Availability of professional development is a prime factor in public health professionals considering leaving their position but ultimately deciding to stay (38). Younger employees in particular cited a lack of advancement opportunities as a prime reason for considering leaving (19, 20). This points to a “greener pastures phenomenon” where younger workers are more willing to change jobs for better opportunities (19, 20).

##### 3.3.2.2 Opportunities

Investing in training and development of existing employees has the benefit of helping to retain employees and encourage them to advance within the organization, with both formal and informal training opportunities forming an important role in professional development (48). Employees with a long tenure at the agency have invaluable institutional knowledge. Some evaluations demonstrated the success of posting certain vacancies for internal candidates only or creating new high-level and lateral positions for internal candidates to broaden employee experience in retaining mid-career and senior-level employees (50). Additionally, studies focused on both state and local health departments suggest that creating internal growth opportunities for employees will assist in preserving this institutional knowledge and will support organizational succession planning (48, 51–53).

Younger generations in particular report that opportunities for advancement hold great value when considering whether to stay with a current public health employer (19). Offering those individuals growth and development opportunities within the agency can help retain internal talent and experience (19, 20). To support this initiative, some agencies have experimented with leadership development programs for high-potential employees. Once high-potential candidates are identified, a systematic approach is adopted for their focused development by recognizing areas of competency enhancements, setting goals, creating individualized development plans, planning strategic job assignments, providing mentoring, and evaluating developmental progress (50, 54). These candidates have also been offered targeted training for advanced roles, provided opportunities to practice new techniques, and offered mentorships to nurture these skills.

## 4 Discussion

In this study, we assessed the current environment of peer reviewed literature related to recruitment and retention, specifically with the goal of identifying opportunities that LHDs can implement in their agencies. Our review highlights the barriers related to attracting and retaining qualified professionals in LHDs. Studies elevated low salaries and perception of unsatisfying work environments as key issues hindering recruitment efforts, particularly among diverse candidates and recent graduates. The opportunities offered in the literature include creating welcoming work environments, offering competitive salaries, and emphasizing the meaningful nature of public health work. Other promising initiatives include academic partnerships, internship programs, and streamlined hiring processes. Retaining employees requires attention to organizational culture, professional development opportunities, and advancement pathways within the agency. Lack of rigorous data and evaluations make it difficult to determine the true extent of workforce barriers and the effectiveness or impact of strategies and opportunities to overcome them.

While the literature we obtained illuminated many barriers and opportunities relevant to the public health workforce, various gaps existed in the literature. Many studies cited are based on limited samples, and few of these studies discussed the external validity of their findings. Additionally, there is limited literature about the specific tested strategies to improve hiring processes and workplace culture, the impact of relying on the meaningful nature of public health work, data related to pay disparities between public health and other employers, and on fostering a diverse workforce. Lastly, literature was further limited in providing tested, concrete ways of addressing these issues. For example, while it was identified in the literature that job seekers prefer proactive and timely communications about the status of the application process (55), documented solutions were limited and not accompanied by data supporting their effectiveness. Similarly, there were no evaluated strategies for improving public health workplace culture, even though this was frequently cited as a point of dissatisfaction for current and potential employees. This is particularly relevant in a post-COVID climate, where state and local government employees’ expectations for hybrid and remote work have shifted, as 60% of employees who currently work remote reported that they would look for other places of employment if their current employer does not offer some type of remote schedule (56). This underscores the need for LHDs to look at applicable, effective private sector practices like hybrid and remote work that enhance LHD culture and make it more appealing (56).

While dissatisfaction with pay was a recurrent point in many articles, it was explored as a general phenomenon contributing to workforce barriers. There was no fully understand the magnitude of the differences between public health salaries and those at competing employers. Such context is important in providing evidence necessary for advocacy to formulate opportunities to this widespread barrier. There is recent work, published since this search was conducted, that has begun to look at this contrast and found wide disparities in pay between the private and public sectors. For example, chief executives make 47% less, emergency managers make 25% less, and epidemiologists make 22% less in local government employment than they do in the private sector (57). Further, governmental agencies operate within budget constraints very different from the private sector. If salary increases are not possible, clearly stating salary ranges in job postings is crucial to allow applicants to make informed decisions about whether or not to apply (14).

Our searches gleaned little peer-reviewed literature focused on diversity in the public health workforce. This is important because the barriers affecting the public health workforce as a whole are likely affecting individuals from marginalized communities to a larger degree. Additionally, these individuals are likely encountering unique barriers. An example that did present in our literature was the benefit of mentoring for all employees, but a formal process should be utilized to prevent biases from being upheld via informal mentoring recruitment. Unpaid internships can present a barrier to students from diverse backgrounds, given that such opportunities favor students with the privilege to forgo paid employment and utilize financial support from family (58). Further, strict degree requirements on job postings may present a barrier for those who have not had the opportunity to complete a formal degree but who offer valuable experience (59). In a field where alleviating racial, ethnic, and socioeconomic health disparities is a priority, importance should be placed on understanding and alleviating these same disparities within our own workforce. More research is needed to fully understand barriers and associated opportunities to support a diverse public health workforce.

Non-peer reviewed sources offer some insight into recruitment and retention strategies that the public health agencies and scholars could consider. These sources on recruitment focus mainly on marketing efforts to increase the appeal of public health employment, and gray literature sources on retention were limited in quantity. To help attract applicants, public health agencies can borrow strategies from the private sector by building an agency brand, reaching potential applicants at many different touch points (including non-traditional avenues like social media and billboards), and reframing positions to sound more appealing (55). In terms of improving the hiring process, recruiters can help overcome a daunting public service application process by selling the appeal of the agency and individual positions before getting into the specifics of the hiring process (55). While such marketing may help get applicants in the door, it is still critical to address retention barriers to get these employees to stay. To this end, “stay interviews” can be conducted with current longtime employees to understand why they stay with the agency and inform positive points that could be highlighted with prospective applicants (60).

### 4.1 Limitations

Although conducted as a literature review, our review was conducted systematically, yet we acknowledge that the main shortcoming of our methods is that the articles were reviewed and screen by only one author and could be subject to erroneous inclusions or exclusion. Additionally, the timeframe of our study prevented inclusion of more current literature related to workforce implications of the COVID-19 pandemic. Our searches were conducted in 2022, when little COVID-19 literature related to the public health workforce had been published. Thus, our findings likely do not capture more current considerations for the public health workforce related to COVID-19 and the fundamental changes that the public health field has experienced due to the pandemic. Finally, full article review and thematic analysis were conducted by one author which could potentially introduce bias in our findings.

### 4.2 Implications for policy and practice

- To address recruiting barriers, health departments can work to create a welcoming environment for entry-level employees, emphasize the impact of public health in marketing efforts, establish partnerships with academic institutions, and create internship programs–preferably paid.
- To address retention barriers, health departments can seek to improve the workplace environment, support management training for public health managers, providing mentoring opportunities, supporting professional development, and succession planning.
- Uncompetitive salaries are a recurrent barrier and one that is difficult to address without significant funding changes. We emphasize that more investment in public health agencies is critical to allow them to offer salaries that can remain competitive with other employers.
- To address gaps in the literature, researchers and health departments alike should seek to further investigate specific strategies to improve public health workplace culture, the extent of salary disparities between public health employers and other employers, and barriers and opportunities to supporting diversity in the public health workforce.

## 5 Conclusion

The public health workforce continues to face staffing shortages, despite the influx of public health degree graduates. The findings from this environmental scan highlight the primary barriers that LHDs face relating to recruitment and retention are non-competitive salaries, a perceived lack of employee autonomy, cumbersome HR protocols, and an unsatisfying environment for a diverse workforce. We provide LHDs with strategies that they can implement to strengthen their workforce. To enhance recruitment, LHDs should market the rewarding aspect of working in governmental public health, establish partnerships with academic institutions, and develop internship programs. To improve retention, LHDs should strengthen the organizational work environment by supporting professional growth, mentoring programs, and succession planning. Further efforts are needed to identify specific strategies for improving workplace culture, quantify the pay disparity between health departments and their competitors, and better understand the barriers and opportunities related to supporting a diverse workforce.

## Data Availability

The raw data supporting the conclusions of this article are proprietary to Cook County Department of Public Health. Further inquiries can be directed to the corresponding author.
