# Supplementary material for: A review of recruitment and retention strategies in U.S. local health departments: insights and practical solutions
Source: Front Public Health. 2025 Jul 17;13:1516027. doi: 10.3389/fpubh.2025.1516027 (PMC12312223; doi:10.3389/fpubh.2025.1516027)
Supplement: Supplementary file 1 [file Data_Sheet_1.docx]

Appendix A: Search Terms and Criteria

| Search Name | Publish or Perish Search Terms |
| --- | --- |
| 01-GS-EL | "public health" (agenc* OR department) (local OR county OR city) workforce (recruit* OR staffing OR hiring) (internship OR practicum OR "underrepresented minor*" OR "recent grad*" OR "entry level" OR "entry-level") (program OR framework OR model) from ... |
| 02-GS-EL | "public health" (agenc* OR department) (local OR county OR city) workforce (recruit* OR staffing OR hiring) (internship OR practicum OR "underrepresented minor*" OR "recent grad*" OR "entry level" OR "entry-level") (outine OR curriculum OR pilot) from ... |
| 03-GS-EL | "public health" (agenc* OR department) (local OR county OR city) workforce (recruit* OR staffing OR hiring) (pipeline OR "pipeline building opportunit*" OR pathway "pathway building opportunit*" OR "interprofessional education") from 2022 to 2002 |
| 04-GS-EL | "public health" (agenc* OR department) (local OR county OR city) workforce (recruit* OR staffing OR hiring) (students OR "school of" OR "young people" OR "young adults" OR "underrepresented minor*" OR "recent grad*") (program OR framework OR model) fro... |
| 05-GS-EL | "public health" (agenc* OR department) (local OR county OR city) workforce (recruit* OR staffing OR hiring) (students OR "school of" OR "young people" OR "young adults" OR "underrepresented minor*" OR "recent grad*") (outline OR curriculum OR pilot) fr... |
| 06-GS-EL | "public health" OR "health department" [title], (local OR county OR city) workforce (recruit* OR staffing OR hiring) (internship OR practicum OR "underrepresented minor*" OR "recent grad*" OR "entry level" OR "entry-level") from 2002 to 2022 |
| 07-GS-EL | "public health" OR "health department" [title], (local OR county OR city) workforce (recruit* OR staffing OR hiring) (pipeline OR "pipeline building opportunit*" OR pathway OR "pathway building opportunit*" OR "interprofessional education") from 2002 t... |
| 01-WoS-EL | "public health" OR "health department" [title], workforce AND "entry level" OR entry-level OR staffing OR hiring OR recruit* AND program OR framework OR model OR ourline OR curriculum OR pilot from 2002 to 2022 |
| 01-PM-EL | "public health" OR "health department" AND workforce AND "entry level" OR entry-level OR staffing OR hiring OR recruit* from 2002 to 2022 |
| 01-GS-MS | "public health" (agenc* OR department) (local OR county OR city) workforce (recruit* OR staffing OR hiring OR retention OR promotion OR development OR pipeline OR pathway) ("mid career" OR "mid-career" OR "mid level" OR "mid-level") from 2022 to 2002, no citations, no patents |
| 02-GS-MS | "public health" (agenc* OR department) (local OR county OR city) workforce (recruit* OR staffing OR hiring OR retention OR promotion OR development OR pipeline OR pathway) ("senior career" OR "senior-career" OR "senior level" OR "senior-level") from 2022 to 2002, no citations, no patents |
| 03-GS-MS | "public health" (agenc* OR department) (local OR county OR city) workforce (experience* OR proficient OR trained OR competent OR qualified) ("mid career" OR "mid-career" OR "mid level" OR "mid-level") from 2022 to 2002, no citations, no patents |
| 04-GS-MS | "public health" (agenc* OR department) (local OR county OR city) workforce (experience* OR proficient OR trained OR competent OR qualified) ("senior career" OR "senior-career" OR "senior level" OR "senior-level") from 2022 to 2002, no citations, no patents |
| 05-GS-MS | "public sector" (local OR county OR city) workforce (recruit* OR staffing OR hiring OR retention OR promotion OR development OR pipeline OR pathway) ("mid career" OR "mid-career" OR "mid level" OR "mid-level") from 2022 to 2002, no citations, no patents |
| 06-GS-MS | "public sector" (local OR county OR city) workforce (recruit* OR staffing OR hiring OR retention OR promotion OR development OR pipeline OR pathway) ("senior career" OR "senior-career" OR "senior level" OR "senior-level") from 2022 to 2002, no citations, no patients |
